# Physiotherapy to improve physical activity in community-dwelling older adults with mobility problems (Coach2Move): study protocol for a randomized controlled trial

**DOI:** 10.1186/1745-6215-14-434

**Published:** 2013-12-17

**Authors:** Nienke M de Vries, J Bart Staal, Steven Teerenstra, Eddy MM Adang, Marcel GM Olde Rikkert, Maria WG Nijhuis-van der Sanden

**Affiliations:** 1 Radboud University Nijmegen Medical Centre, Scientific Institute for Quality of Healthcare (IQ healthcare), P.O. Box 9101, 114 (IQ healthcare), Nijmegen 6500, HB, The Netherlands; 2 Department for Health Evidence (HEV), Radboud University Nijmegen Medical Centre, P.O. Box 9101, 138 (HEV), Nijmegen 6500, HB, The Netherlands; 3 Department of Geriatrics, Radboud University Nijmegen Medical Centre, P.O. Box 9101, 925 (Dept Geriatrics), Nijmegen 6500, HB, The Netherlands; 4 Scientific Institute for Quality of Healthcare (IQ healthcare), Radboud University Nijmegen Medical Centre, P.O. Box 9101, 114 (IQ healthcare), Nijmegen 6500, HB, The Netherlands

**Keywords:** Physiotherapy, Frailty, Physical activity, Mobility

## Abstract

**Background:**

Older adults can benefit from physical activity in numerous ways. Physical activity is considered to be one of the few ways to influence the level of frailty. Standardized exercise programs do not necessarily lead to more physical activity in daily life, however, and a more personalized approach seems appropriate. The main objective of this study is to investigate whether a focused, problem-oriented coaching intervention (‘Coach2Move’) delivered by a physiotherapist specializing in geriatrics is more effective for improving physical activity, mobility and health status in community-dwelling older adults than usual physiotherapy care. In addition, cost-effectiveness will be determined.

**Methods/Design:**

The design of this study is a single-blind randomized controlled trial in thirteen physiotherapy practices. Randomization will take place at the individual patient level. The study population consists of older adults, ≥70 years of age, with decreased physical functioning and mobility and/or a physically inactive lifestyle. The intervention group will receive geriatric physiotherapy according to the Coach2Move strategy. The control group will receive the usual physiotherapy care. Measurements will be performed by research assistants not aware of group assignment. The results will be evaluated on the amount of physical activity (LASA Physical Activity Questionnaire), mobility (modified ‘get up and go’ test, walking speed and six-minute walking test), quality of life (SF-36), degree of frailty (Evaluative Frailty Index for Physical Activity), fatigue (NRS-fatigue), perceived effect (Global Perceived Effect and Patient Specific Complaints questionnaire) and health care costs.

**Discussion:**

Most studies on the effect of exercise or physical activity consist of standardized programs. In this study, a personalized approach is evaluated within a group of frail older adults, many of whom suffer from multiple and complex diseases and problems. A complicating factor in evaluating a new approach is that it may not be automatically adopted by clinicians. Specific actions are undertaken to optimize implementation of the Coach2Move strategy during the trial. Whether or not these will be sufficient is a matter we will consider subsequently, using quality indicators and process analysis.

**Trial Registration:**

The Netherlands National Trial Register: NTR3527.

## Background

Physical activity is considered one of the few ways to influence frailty in older adults [1-4]. The extent to which the level of frailty can be influenced by physical activity remains unclear, however. Frailty is defined as a complex state of increased vulnerability to adverse health outcomes [5]. The causes as well as the consequences of frailty are diverse and originate from multiple dimensions (physical, psychological and social), which makes frailty a difficult concept to measure [6]. A small number of studies have shown a positive effect of physical activity on the level of frailty by measuring physical aspects of frailty [7,8]. The positive effect of exercise on other frailty-related aspects of health, for example, mood and cognition, has also been confirmed in studies [9,10]. The complex interaction between the physical, psychological and social dimensions of frailty influences the level of frailty. The total level of frailty is, therefore, not equivalent to the sum of its components. When, for example, muscle strength improves because of an exercise intervention, this could lead to better mobility and the ability to become more physically active. Also, it may become easier to go outdoors, which can positively influence mood and social interaction. As a consequence, when all these dimensions are taken into account, the level of frailty may improve by even more than the levels of the separate components. The multidimensional level of frailty, considering all aspects of frailty in interaction using one measurement instrument, has not been evaluated in intervention studies on exercise and physical activity hitherto.

Furthermore, while the positive effect of physical activity on different aspects of health is widely accepted, many older adults remain sedentary [11]. Standardized supervised exercise interventions do not necessarily increase the level of physical activity in daily life [12]. It is a great challenge to attain the behavioral change that is necessary to become more physically active in the long term and to improve adherence to physical activity programs or interventions. Individually adapted programs that aim for participants to become more physically active in daily life are probably more (cost-) effective [13]. We have developed an individual intervention aimed at promoting physical activity in the broad population of older adults suffering from or at risk of mobility problems. Most older adults have to deal with multiple and complex problems and diseases, and yet often they do not fulfil the specific inclusion criteria used in clinical studies. Therefore, we developed an intervention aimed at a very broad population of older adults. Because of the complexity of this intervention, the Medical Research Council (MRC) framework was used as a guide in developing the intervention. The MRC framework consists of five successive phases: preclinical or theoretical phase (phase I), modelling phase (phase II), exploratory trial (phase III), randomized controlled trial (phase IV), and long-term implementation (phase V). In the theoretical phase, we performed two systematic reviews. The first review deals with instruments for measurement of frailty. In this review, we concluded that numerous measurement instruments exist, but none of them has been developed as an outcome measure [6]. Therefore, we subsequently developed an outcome instrument on frailty; the Evaluative Frailty Index for Physical Activity (EFIP) [14]. In a second article, we review the literature on the effect of physical exercise therapy in older adults who have problems with physical functioning and/or have to deal with co-morbidity [12]. The included literature was evaluated in meta-analyses and we concluded that mobility and physical functioning are positively influenced by physical exercise therapy. Strength training also seemed to be of great importance and a personalized approach may result in long-term positive effects (>12 months). No positive effects on quality of life and the level of physical activity could be found, which confirms that an exercise intervention does not necessarily bring about changes in level of physical activity.

In the modelling phase of the MRC framework, we developed the intervention based on the findings from the theoretical phase and expert consultation. This process will be described in detail in another publication. The intervention was called ‘Coach2Move’ and is based on clinical reasoning conforming with the Hypothesis Oriented Algorithm for Clinicians (HOAC)-II [15,16] and the International Classification of Functioning, Disability and Health (ICF) [17]. The HOAC-II is an algorithm for clinical reasoning that describes all steps to be taken in order to make appropriate decisions about the treatment of patients. The steps of the HOAC-II were specified for the physiotherapy treatment of frail older adults with mobility problems in the Coach2Move strategy. The core elements of the Coach2Move strategy are: 1) increasing the level of physical activity and (social) participation; 2) patient-identified goals, and thereby improving adherence; 3) enablement instead of disablement (what a patient can do instead of what a patient cannot do); and 4) self-management. The Coach2Move strategy was tested for feasibility and efficacy in a pilot study (NM De Vries *et al*., unpublished observations). The results of this pilot study showed that the Coach2Move strategy is well appreciated by both physiotherapists and patients and that a positive effect on physical activity, mobility, frailty and quality of life can be expected. The present article describes the study design of a Randomized Controlled Trial (RCT) to evaluate the Coach2Move strategy. The main objective of this RCT is to test the hypothesis that the Coach2Move strategy delivered by a physiotherapist specializing in geriatrics is more effective in improving physical activity in community-dwelling older adults than is usual physiotherapy care. As a secondary objective, the effect of the Coach2Move strategy on the (multidimensional) level of frailty, mobility and quality of life, as well as cost-effectiveness, will be assessed in comparison with usual physiotherapy.

## Methods/Design

### Study design

The study design is a single-blinded randomized controlled trial (RCT) in which the consort guidelines are being followed. The RCT is being performed in thirteen physiotherapy practices. In each participating practice both a general physiotherapist (PT) and a PT specializing in geriatrics (GPT) will participate in the study. Participating GPTs will be trained in the Coach2Move strategy. The PTs will provide the usual care.

### Participants

The population consists of (pre-)frail older adults with mobility problems and/or a physically inactive lifestyle who are at risk of loss of mobility in the near future. This study focuses on elderly people living relatively independently at home or in a home for elderly people. Older adults with acute health problems for which hospital admission or admission to a nursing home is necessary are excluded. Older adults signed up for physiotherapy are considered for inclusion, whether referred by a physician or having decided to attend physiotherapy by themselves.

The inclusion criteria are as follows:

1. Older adults aged ≥ 70 years living independently at home or in an older persons’ home.

2. A mobility problem and/or a physically inactive lifestyle (< 30 minutes per day) rated as such by the participant, their relatives or the referring physician.

The exclusion criteria are as follows:

1. Unable to walk 5 meters (waling aid allowed).

2. Unable to follow verbal or written instructions, operationalized by a minimum score of 21 points on the Minimal Mental State Examination (MMSE), or unable to understand Dutch.

3. Palliative phase of illness.

4. Acute illness with hospital indication.

5. Severe degenerative neurological illness.

6. Having a contraindication for being physically active.

7. Having had physiotherapy for a period longer than four weeks during the last six months.

### Randomization, blinding and treatment allocation

All older adults (≥70 years old) who sign up for physiotherapy in one of the participating physiotherapy practices, because of mobility problems and/or physical inactivity are asked to participate in the trial by either a physiotherapist or a practice secretary. If willing to participate, they are called by a member of the research team within two days. The member of the research team will determine potential eligibility by telephone and make an appointment for baseline measurement. An informed consent form is signed by both the participant and the research assistant before the baseline measurement. The final decision on inclusion or exclusion and randomization takes place after baseline measurement. Randomization is done on the individual level through a computer-generated random-sequence table. Pre-stratification is applied by physiotherapy practice. Opaque, sequentially numbered, sealed envelopes are prepared for each stratum (that is, physiotherapy practice) by a researcher who is not involved in enrolling the participants, in assigning them to their groups or performing follow-up measurements. Each of the envelopes contains a sheet of paper indicating one of the two interventions. The sealed envelopes are delivered to the participating physiotherapists or practice secretaries after baseline measurement. Participants learn their group assignments after the researcher or research assistant involved in the baseline measurement has left. Follow-up measurement at three and six months is performed by two research assistants who are unaware of group allocation.

### Informed consent and ethical approval

All eligible patients are informed about this study and given the time they needed to consider participation. The investigator of this study and an independent physician not involved in the study may be approached for questions. Patients who are willing to participate sign an informed consent form. This study has been approved by the medical ethical review board of the Radboud University Medical Centre, Nijmegen (registration number: 2012/233), and is registered in The Netherlands National Trial Register (registration number: NTR3527).

### Intervention

The intervention consists of geriatric physiotherapy according to the Coach2Move strategy ([see Additional file 1 and Table 1]). The Coach2Move strategy helps the GPT in clinical reasoning by providing an extensive, pre-structured and systematically organized diagnostic protocol. For the diagnostic phase of the Coach2Move strategy more time (1.5 hours) is available than for the conventional physiotherapy intake (30 minutes). Impairments and disabilities, but also possibilities, wishes, barriers and facilitators relevant to physical functioning are thoroughly examined. Motivational interviewing is used to find and deal with the barriers to individuals becoming physically active, but also to find out what personal goals a patient wants to achieve. Physiotherapy treatment in the Coach2Move strategy is focused on increasing motivation to be physically active by working on personal goals and removing physical barriers. Using shared decision making together with the patient, the GPT sets SMART goals – specific, measurable, acceptable, realistic and (with a) timeline. The GPT coaches the patient in reaching and maintaining his/her own goals (self-management) using appropriate feedback.

**Table 1:** The innovative elements of the Coach2Move strategy

**The innovative elements of the Coach2Move strategy**	
1	Use of motivational interviewing: exploring questions for help and barriers and facilitators in relation to physical activity.
2	Use of an algorithm (HOAC-II*^a^) that emphasizes an extensive intake and supports clinical reasoning in order to set priorities.
3	Shared decision making on meaningful treatment goals to increase physical activity.
4	Coaching on self-management to increase long-term results.
5	Focusing on meaningful activities at home with help from family, friends or professionals.
6	Working according to three patient-tailored intervention profiles with a predefined number of sessions.

^a^HOAC, Hypothesis Oriented Algorithm for Clinicians.

In Coach2Move, three intervention profiles with a predefined number of sessions can be chosen. Patients are categorized into one of these three profiles based on the complexity of their problems and their potential for improvement. The first profile deals with patients who do not need physiotherapy intervention on the level of body function or structure, but who just need coaching and advice (≤4 sessions). The second profile is aimed at improving functions, abilities and activities over a course of seven to nine sessions for patients who need temporary treatment to overcome barriers to becoming physically active. In the third profile, patients with specific problems in participation, activities and functions are treated and coached in 12 to 18 sessions.

GPTs are trained in the Coach2Move strategy in a basic training of two days. This training focuses on:–

1. Clinical reasoning.

2. Using appropriate measurement instruments in the diagnostic phase but also in the evaluation of the intervention and as feedback instrument for the patient and therapist.

3. Shared decision making.

4. SMART focused goals (specific, measurable, acceptable, realistic, timeline).

5. Learning to focus on coaching and self-management.

6. Learning to work in a patient-centered way and using measurements for feedback.

7. Learning motivational interviewing skills.

8. Learning skills for adequate coordination with other formal and informal caregivers involved.

In this study, the control intervention consists of conventional physiotherapy provided by a PT. A one-day basic training is organized for the PTs to enhance their motivation to participate in this RCT. The training consists of general information regarding the treatment of older adults. In this training, no attention is paid to clinical reasoning, measurement instruments, shared decision making, motivational interviewing or self-management.

The treatment delivered by both the GPTs and the PTs consists of conventional physiotherapy modalities such as training activities in daily living, balance and muscle strength. No standardized intervention is prescribed and in both groups the physiotherapist is free to determine the appropriate intervention and level to reach the intervention goals. We expect less focus on physical activity in the control group. Moreover, the control group can freely decide how many sessions are needed. This is standard procedure in physiotherapy care in the Netherlands.

The key elements of the Coach2Move strategy are set out in Table 1 and these are the main distinguishing factors between the intervention and the control group. Even though shared decision making, patient-centered treatment and goal setting on participation level are considered important, physiotherapists have difficulty actually applying this in clinical practice [18-20]. The Coach2Move strategy offers education in these elements and supports physiotherapists during the implementation, which we expect to result in a contrast between the intervention and control groups. Both therapies are expected to be effective; however, we hypothesize that the Coach2Move strategy will be more effective on the level of physical activity and in reaching treatment goals in fewer physiotherapy sessions.

### Study parameters

The main study parameter is the level of physical activity during follow-up over six months. The level of physical activity will be measured using the LASA Physical Activity Questionnaire (LAPAQ) [21]. The LAPAQ is a reliable and valid instrument specifically developed for older adults. The LAPAQ is a comprehensive questionnaire on diverse (physical) activities of daily living including walking, riding a bicycle, gardening, light household chores, heavy household chores and sporting activities. Based on the results of this questionnaire, the average amount of physical activity in minutes per day and minutes per week can be assessed.

Secondary study parameters include mobility, quality of life, level of frailty, fatigue and perceived effect. Mobility is measured using the ‘get up and go’ test (GUG) [22], the six-minute walking test (6MWT) [23] and walking speed (WS) timed over a 10-meter distance [24]. During the modified GUG, the patient is instructed to rise out of a chair and walk 10 meters [24]. This procedure is timed and the duration in seconds is the score for this test. The effort of completing the same test is measured using the BORG scale of perceived exertion [25]. The Borg scale ranges from 6 to 20, where 6 indicates no exertion and 20 the highest possible exertion. The 6MWT consists of six minutes walking at the patients’ self-determined pace. The score on this test is the distance in meters covered in six minutes. WS is timed on a 10-meter distance. The mean time in seconds it takes the patient to walk this distance (at his/her own preferred speed) over three tries, is the final score on this test [24]. The SF-36 is used as a measure of quality of life [26]. The SF-36 is a 36-item questionnaire on health status. Questions relate to eight domains: vitality, physical functioning, bodily pain, general health perceptions, physical role functioning, emotional role functioning, social role functioning and mental health. Each domain is equally weighted and a standardized scoring system results in two scores: a physical score and a mental/emotional score. Both scores range from 0 to 100 with an average score of 50, and a higher score indicating a better quality of life.

To measure the level of frailty, the Evaluative Frailty Index for Physical activity (EFIP) is used [14]. The EFIP is a 50-item questionnaire based on deficit accumulation (symptoms, signs, disabilities) in multiple domains (physical, psychological, social and general health status). The score on this questionnaire is expressed as the ratio of deficits present to the total number of deficits considered. The score ranges from 0 to 1, with 0 indicating no frailty and 1 indicating maximum frailty.

Fatigue is measured using the Numeric Rating Scale (NRS)-fatigue [27], which is a scale from 0 to 10 in which the patient can indicate what level of fatigue is experienced. A score of 0 represents no fatigue at all and a score of 10 represents the most possible fatigue.

Perceived effect is measured with the PSC (patient specific complaints) questionnaire and Global Perceived Effect (GPE). The PSC is an instrument in which patients can determine three activities in which they experience the most trouble [28]. For each activity one can judge the level of experienced trouble on a scale from 0 to 10 in which 0 indicates no trouble at all and 10 indicates that the activity cannot be performed. The Global Perceived Effect (GPE) [29] score is used to measure the patient’s opinion about the effect of the intervention on a nine-point scale ranging from very much improvement to very much deterioration.

Co-morbidity is registered using the Cumulative Illness Rating Scale-Geriatrics (CIRS-G) [30]. The CIRS-G in combination with registration of the physiotherapy intervention during each session and registration of health care utilization will be used to explore whether a physiotherapy intervention is adjusted because of the presence of co-morbidity, if the presence of co-morbidity influences the effect of an intervention, and if co-morbidity brings about interventions other than physiotherapy.

Health care utilization is registered by means of a questionnaire. The questionnaire considers the type and number of medical consultations, medication use, hospital stay, nursing home stay, use of residential facilities, professional home care and the purchase of assistive devices in the three months before the questionnaire is taken.

All these measurements are performed at baseline, at three months and at six months by research assistants who are blind regarding the group allocation of individual patients. At the final measurement, patients will also be asked about their adherence during the physiotherapy process by means of a short questionnaire.

An influencing variable is the quality of the Coach2Move strategy as delivered by the participating GPTs. Therefore, we will provide the GPTs with an electronic patient file in which the clinical reasoning process is supported. An independent researcher, not involved in follow-up measurement (NV), will have access to the patient files of all participating GPTs and check the records for consistency with the Coach2Move strategy. If the Coach2Move strategy is not being sufficiently followed, the GPT concerned will be coached by the independent researcher in the implementation of the strategy for that specific patient. We are also developing quality indicators to research fidelity to the actual Coach2Move strategy by the participating GPTs. PTs register their physiotherapy process in the usual way and are not coached during the intervention. The patient files of the PTs will be scored with the quality indicators at the end of the trial to determine the amount of contrast between the GPT and PT groups.

### Patient flow

**Figure 1:**
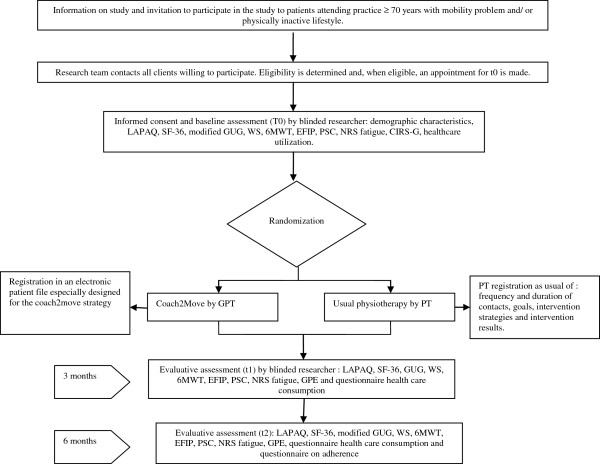
**Patient flow.** CIRS-G, cumulative illness rating scale for geriatrics; EFIP, evaluative frailty index for physical activity; GPE, global perceived effect; GPT, geriatric physical therapist; LAPAQ, LASA physical activity questionnaire; modified GUG, modified get up & go test; NRS fatigue, numeric rating scale fatigue, PSC, patient specific complaints; PT, physiotherapist; SF-36, short form 36; WS, walking speed; 6MWT, 6 minute walking test.

Figure 1 describes the patient flow. This RCT has an inclusion period of 15 months and a follow-up of six months. This means that the RCT will be completed in 21 months. Patient recruitment started in September 2012 and will end in November 2013.

### Sample size

To calculate the sample size, we use the formula for an ANCOVA, derived by Teerenstra *et al*., which states that the sample size uncorrected for clustering and baseline measurement has to be multiplied by two factors [31]. First, a factor that accounts for the clustering: DE = (1 + (n-1) *ICC), where n is the average number of patients per therapist and ICC is the intra-cluster correlation of patients within a therapist. Second, a factor that accounts for the adjustment for baseline: (1-r^2^) with r = r_c*(n*ICC/DE) + r_s*((1-ICC)/DE), where r_c is the autocorrelation and r_s is the autocorrelation of patients. The autocorrelation is the correlation between the three-month measurement and baseline measurement.

#### *Uncorrected sample size*

In our pilot study of ten patients treated by one of two therapists, we found on log scale an increase of 1.21 from baseline and a standard deviation of 1.0 (in the pre-intervention scores). This corresponds to a 3.35 fold increase from baseline scores on the untransformed LAPAQ scale. The control group in our study is comparable to the experimental group in the study performed by Rubenstein *et al*., which found a 20% increase from baseline (1.2-fold increase) [32]. Thus the intervention is estimated to be 3.35/1.2 = 2.8 times as effective as the control treatment. To account for possible overestimation of the effect by the pilot study and possible contamination, we assume that at least 60% of this effect will be retained; that is, the intervention is at least 1.68 times as effective as the control. Then, the effect on log scale is 0.5 (sd = 1.0 as above). The uncorrected sample size to detect this difference with 80% at a significance level of 0.05 is 64 patients per group.

#### *Adjustment for baseline*

From our pilot study we estimated a subject-autocorrelation r_s = 0.73; therapist-autocorrelation could not be estimated (too few therapists), but conservatively we set it to be moderate r_c = 0.4. Then r = 0.6 and the correction for baseline adjustment is 1-(0.6^2^) = 0.64.

#### *Clustering*

In our pilot study we found ICC = 0 and, conservatively setting the ICC = 0.05, the correction for clustering is 1 + (n-1)*0.05. Allowing for maximal 20% dropout, the corrected sample size can be calculated with: 64*0.64*(1 + (n-1)*0.05)* 1/(80%). Table 2 gives the sample sizes when a different number of physiotherapy practices are included. If each participating physiotherapist (2 per practice, 13 practices = 26 physiotherapists, 12 physiotherapists per group) were to treat 5 patients, a total number of 130 patients would be included (65 patients per group). This is more than the 62 patients per group necessary according to the power calculation (see Table 1). If each therapist were to treat four patients, however, the power would not be reached. Therefore we will include 65 patients per group (total = 130) in this trial.

**Table 2:** Sample size calculation

**n = Number of patients per cluster**	Sample size per group: 64*0.64*(1 + (n-1)*0.05)* 1/(80%)	**#Clusters group: sample size /n**
10	75	8
9	72	8
8	70	9
7	67	10
6	64	11
5	62	13
4	59	15

### Statistical analysis

Descriptive statistics will be used to present group characteristics on age, education, marital status, multi-morbidity, level of frailty, number of post-operative patients, therapy adherence and adverse effects. Any asymmetry between the intervention and control group on these variables at baseline will be statistically corrected. The effects of the intervention are analyzed according to the intention-to-treat principle. The analysis of the primary and secondary outcome measures employed a linear mixed model to relate the three- and six-month outcomes to the effect of the treatment. Therapist is taken as a random effect in this model to account for the clustering of patients with therapists. Measurement (baseline, 3 months, 6 months) is included as a random effect to account for correlation of measurements over time. Because this analysis uses all three measurements (baseline, 3 and 6 months), it will actually be more powerful than an ANCOVA analysis and the power of the study will be more than 80%.

#### Economic evaluation

This study investigates the potential efficiency of the Coach2Move strategy compared with usual physiotherapy care in frail elderly patients attending physiotherapy after referral by a physician or by self-referral. The economic evaluation is based on the general principles of a cost-utility analysis, applying a health care perspective. Primary outcome measures for the economic evaluation are: costs and quality adjusted life years (QALY) (SF-6D, part of SF-36) [26]. The incremental cost-effectiveness ratio (ICER) ‘cost per QALY gained’ will be computed and uncertainty surrounding this ICER will be determined using a non-parametric bootstrap method. A cost-effectiveness acceptability curve will be derived that is able to evaluate efficiency by using different thresholds (Willingness To Pay) for a QALY gained. The impact of uncertainty surrounding deterministic parameters on the ICER will be explored using one-way sensitivity analyses on the range of extremes.

#### *Cost analysis*

The cost analysis measures on patient level, volumes of care (consisting of the production factors: personnel, materials and capacity in a prospective way using therapist scoring sheets (CRFs)). Per arm full cost-prices will be determined using standard unit cost-prices according to the Dutch guidelines for costing research [33]. If for certain procedures standard unit cost-prices are not available, real cost-prices will be determined using activity based costing. This approach applies to the health care production part of the economic evaluation. Medical costs will be estimated using a questionnaire on a three-months recall basis in line with the follow-up pattern of the clinical trial [34,35]. Non-health care costs, like productivity losses, seem irrelevant for this population

#### *Health status*

To measure the quality of the health status of the patients the SF-36 will be used. The SF-36 is a widely used measure of general health in clinical studies throughout the world. It currently generates eight dimension scores and two summary scores for physical and mental health. The SF-6D [34] provides a means for using the SF-36 in economic evaluation by estimating a preference-based single index measure for health from these data using general population values. The SF-6D allows the analyst to obtain QALYs from the SF-36 for use in cost utility analysis. Utilities will be collected alongside the clinical trial as described earlier in the section ‘Design’. These utilities will be transformed into QALYs using the trapezium method.

## Discussion

### Strengths

This is one of the first RCTs to evaluate a systematic, individually-tailored physiotherapy intervention for older adults embedded in clinical practice. In most research, standardized exercise programs are evaluated while in daily clinical practice physiotherapy usually is a tailor-made intervention. We expect a larger effect from a tailored intervention than from a standardized exercise program. In addition, most other studies aim at a specific population of older adults, for example, older adults with diabetes or arthritis, excluding older adults with multiple comorbidity. However, most older adults have to deal with more than one health problem and do not fulfil the inclusion criteria set in most studies on standardized exercise programs. The intervention studied in this RCT is therefore more representative for clinical practice than the interventions in previously performed RCTs.

### Weaknesses

Although we expect a large effect from an individually tailored intervention, researching physiotherapy also brings about some difficulties because of the variety in the intervention. Every physiotherapist deals with patients in his or her own way. Additionally, because we include a very broad population of older adults in this RCT, there will be some variety in the participants. Finally, implementing a new treatment strategy like Coach2Move is a challenging process. A new strategy is not automatically adopted by healthcare professionals even after they have been trained in that strategy. Various determinants either positively or negatively influence the implementation process, for example, the characteristics of the new strategy, characteristics of the adopting person, characteristics of the organization or characteristics of the socio-political context [36]. Also, healthcare professionals are thought to go through four stages in an implementation process. The first is the dissemination stage in which the professional finds/is supplied with the new strategy. The second is the adoption stage in which the professional develops either positive or negative intentions about the intervention. In the subsequent implementation phase, the professional tries the new strategy and finds out what working with the new strategy means. In the fourth and final stage, the continuation stage, the new strategy becomes part of routine practice or it does not [37]. In this RCT on the Coach2Move strategy, we work with GPTs who have volunteered to participate in this trial and who are willing to change their professional behavior. They did not, however, have the opportunity to ‘practice’ with the Coach2Move strategy before the trial started. Also, willingness to change on the personal level does not mean that the organization (a physiotherapy practice) fully supports implementation of a new strategy. We have tried to improve adherence to the Coach2Move strategy by developing an electronic patient file that supports the GPT in executing the strategy. In addition, the treatment given to each patient in the intervention group is continuously evaluated by one of the researchers (NV), and the GPTs are coached in working according to the Coach2Move strategy. However, when the RCT has ended we still need to consider whether or not implementation has been sufficient. We will use quality indicators and process analysis to do this.

### Ethical approval

This study has been approved by the medical ethical review board of the Radboud University Medical Centre (registration number: 2012/233), Nijmegen, the Netherlands.

## Trial status

Enrollment into the study started on 1 September 2012. Recruitment is expected to be completed by the end of November 2013.

## Abbreviations

CIRS-G: Cumulative illness rating scale-geriatrics; EFIP: Evaluative frailty index for physical activity; GPE: Global perceived effect; GPT: Geriatric physiotherapist; GUG: ‘Get up & go’ test; HOAC: Hypothesis oriented algorithm for clinicians; ICER: Incremental cost-effectiveness ratio; ICF: International classification of functioning, disability and health; LAPAQ: LASA Physical activity questionnaire; MMSE: Mini mental state examination; PSC: Patient specific complaints; PT: Physiotherapist; QALY: Quality adjusted life years; RCT: Randomized controlled trial; WS: Walking speed; 6MWT: Six-minute walking test.

## Competing interests

There are no financial or nonfinancial competing interests to declare in relation to this manuscript by any of the authors.

## Authors’ contributions

NV, BS and RN conceived and designed the study. ST and EA participated in the design of the study, the sample size and power calculations, and developed the plan for statistical analysis of trial outcomes. NV is responsible for trial management, recruitment and data collection. NV drafted the manuscript with contributions from all other authors. All authors read and approved the final manuscript.

## Supplementary Material

Additional file 1 Coach2Move Strategy. Click here for file
