# Supplementary material for: Physiotherapy to improve physical activity in community-dwelling older adults with mobility problems (Coach2Move): study protocol for a randomized controlled trial
Source: Trials. 2013 Dec 17;14:434. doi: 10.1186/1745-6215-14-434 (PMC3878551; doi:10.1186/1745-6215-14-434)
Supplement: Additional file 1 — Coach2Move Strategy. [file 1745-6215-14-434-S1.doc]

**Additional file 1: Coach2Move S**trategy

| **Indication for geriatric physiotherapy consultation:** Actual mobility problems and/ or physical inactivity or expected in the near future (within 3 months) |
| --- |

| **Patient- identified problems (PIPs)** |  |
| --- | --- |
| Enablement: present and desired activities and roles | Disablement: health status, problems in body function and structures, activity, participation, environment and personal problems including problems identified by (informal) caregiver |
| **Non- patient identified problems (NPIPs):**Anticipated problems identified by physiotherapist/ (informal) caregiver/ professional | |
| **Measurement instruments:** LASA Physical Activity Questionnaire (LAPAQ) | |

| - **Present and desirable situation according to ICF classification** - **Hypotheses and examination strategy → consultation other disciplines?** |
| --- |

| **Physiotherapy Exam**  Body Function and Structure: When a problem is expected the following measurement instruments could be used: strength: grip strength & MRC (or 10RM), endurance: 6 minute walking test (if not possible: 2 minute walking test), balance: Berg Balance Scale (BBS)(if not possible Tinitti) & Timed Up& Go Test (TUG), joint mobility: Range Of Motion (ROM), sensory function: examination sensory function, pain: NRS  Activity and Participation: Detailed observation of problematic activities under the normal circumstances for the patient (preferably at home). Measurement instruments: Patient Specific Complaints (PSC) |
| --- |

| **Adjust present and desirable situation according to ICF classification based on physiotherapy examination** |
| --- |

| **Analyses of hypotheses** Analysis whether or not the initial hypotheses are supported by the physiotherapy examination and if there is a relationship between the problems identified. |
| --- |

| **Conclusion/ physiotherapy diagnosis** in ICF terms |
| --- |

| **No intervention, consultation other discipline, geriatric physiotherapist, general physiotherapist.** |
| --- |

| **Profile classification** 1. no mobility problems, but contextual or personal factors making it difficult to maintain physically active. 2. Mobility problems which need short term physiotherapy to be able to maintain a physically active lifestyle safely without guidance. 3. Problems which make a physically active lifestyle at this point impossible. |
| --- |

| **Treatment plan** Goal (SMART), strategy, evaluation plan, treatment contract with patient |
| --- |

Con- sulting other disci-plines?

| **Intervention profile 1**  Increasing the level of physical activity | Information and advice (max 4 sessions) |
| --- | --- |
| **Intervention profile 2**  Temporary intervention to make it possible to safely maintain an active lifestyle without guidance | Information and advice, personal possibilities and restrictions, improving body function and structures (7-9 sessions) |
| **Intervention profile 3**  Improving/ maintaining mobility, activities or participation. | Improving mobility and/ or reducing specific problems in activities, functions and structures: strength, endurance, balance, ROM, pain. (12-18 session) |

| **Treatment evaluation** Continuous evaluation using the measurement instruments that were used in the diagnostic phase |
| --- |
